# Impact of the COVID-19 Pandemic on Outpatient Service in Primary Healthcare Institutions: An Inspiration From Yinchuan of China

**DOI:** 10.34172/ijhpm.2021.119

**Published:** 2021-08-31

**Authors:** Lu Xu, Lin Zhuo, Jie Zhang, Wu Yang, Guozhen Liu, Siyan Zhan, Shengfeng Wang, Huijie Xiao

**Affiliations:** ^1^Department of Epidemiology and Biostatistics, School of Public Health, Peking University, Beijing, China.; ^2^Research Center of Clinical Epidemiology, Peking University Third Hospital, Beijing, China.; ^3^Maternal and Child Health Care Hospital of Ningxia Hui Autonomous Region (Children’s Hospital of Ningxia Hui Autonomous Region), Ningxia Hui Autonomous Region, China.; ^4^Peking University Health Information Technology Co. Ltd, Beijing, China.; ^5^Center for Intelligent Public Health, Institute for Artificial Intelligence, Peking University, Beijing, China.; ^6^Department of Paediatrics, Peking University First Hospital, Beijing, China.

**Keywords:** COVID-19, Outpatients, Primary Healthcare, Utilization, Temporal Trend

## Abstract

**Background:** The coronavirus disease 2019 (COVID-19) pandemic has posed a great challenge to the healthcare system. This study evaluated the impact of the pandemic on the utilization of primary healthcare (PHC).

**Methods:** The outpatient data from 158 PHC institutions in Yinchuan from May 1, 2017 to April 30, 2020 were used. The difference in difference (DID) model was used to analyze the difference in the number of outpatient visits per day, total outpatient expenditure per day, and outpatient expenditure per visit between December 2019 and February 2020 compared with the same periods in two previous years. The autoregressive integrated moving average (ARIMA) modelling was used to investigate the association between the outpatient volume and the number of the last week’s new COVID-19 cases in Yinchuan, Ningxia, and China.

**Results:** From December 2019 to February 2020, the decline in the number of outpatient visits per day (DID: -367.21 times, *P*=.004) was larger than that in two previous years, and a similar trend can be seen in the outpatient expenditure per day. However, the rise in the outpatient expenditure per visit (DID: 19.06 thousand yuan, *P*=.003) was larger than that in two previous years. In 2020, the outpatient visits for most types of diseases decreased from week 3 and rebounded after week 5. The decline and rebound of outpatient visits in the population aged 45 years and older were steeper than in those younger. The outpatient volume was negatively associated with the number of the last week’s new COVID-19 cases.

**Conclusion:** This study indicated a significant impact of the pandemic on PHC service utilization. Since PHC service is the foundation of the healthcare system in most developing countries, measures should be taken to make PHC help cope with the crisis and relieve the burden of hospital care.

## Background

**Key Messages**

** Implications for policy makers**

- Similar to studies on hospital service utilization from other countries, this study also observed obvious avoidance in primary healthcare (PHC) during the coronavirus disease 2019 (COVID-19) pandemic.
- Compared to other types of diseases, the reduction of the PHC utilization for the disease of the respiratory systems was largest, and the rebound for it was six weeks later than most other types of diseases, suggesting that the prevention and control measures recommended during the pandemic could also help curb the spread of other infectious respiratory diseases.
- The decline and rebound of the outpatient service utilization in the middle-aged and elderly population were steeper than in those younger; therefore, vulnerable populations such as the older population should be given extra and distinct attention in the formulation of the emergency strategy.
- Well-prepared PHC and protection guidance for the public are needed to avoid preventable adverse clinical outcomes during the crisis.

** Implications for the public**

The public should have access to necessary primary healthcare (PHC) during the COVID-19 pandemic, since delayed healthcare utilization could cause disabilities and deaths which are preventable. It was reported that during the pandemic, in-person healthcare utilization would not increase the risk of infection if it was safely done; therefore, the public should be relieved to obtain the necessary healthcare in time to avoid adverse clinical outcomes. This study found a greater impact of the pandemic on the middle-aged and elderly population; however, timely healthcare utilization for this population is particularly important due to the high prevalence of chronic diseases.

In December 2019, coronavirus disease 2019 (COVID-19) caused by severe acute respiratory syndrome coronavirus 2 appeared in Wuhan, China.^1^ Gradually, it spread all over the world, leading to a global pandemic.^2^ Similar to other epidemics in the past decades such as severe acute respiratory syndrome,^3^ Ebola^4^ and Middle East respiratory syndrome,^5^ COVID-19 pandemic has a significant impact on healthcare utilization.^6-13^ For example, studies from the USA found a dramatic decrease in utilization of preventive care, utilization of many elective procedures, and hospital admissions during the first two months of the COVID-19 pandemic (ie, March and April 2020).^6,7,10^ A cross-sectional telephone survey in Hong Kong of China reported that 30.4% of the public chose to avoid medical consultations during the initial months of the COVID-19 pandemic.^8^ In mainland China, based on bank card transactions, Zhang et al^12^ evaluated the influence of the COVID-19 pandemic on healthcare expenditure and utilization and found a considerable reduction of total healthcare expenditure and utilization when the pandemic reached its peak. However, previous studies in this regard mainly focused on hospital visits or admissions or failed to distinguish patients’ treatment purposes.

 In developing countries, including China, primary healthcare (PHC) is a cost-effective way to achieve universal health coverage by improving population health outcomes and lowering all-cause mortality,^14,15^ since PHC can provide a wide range of care that can satisfy the majority of a population’s health needs and to meet evolving needs at the community level.^15^ More importantly, the adaptability of PHC contributes to its responsiveness and resilience, especially in face of crisis.^15^ Although PHC is an essential component of the healthcare system, previous studies, most of which were from developed countries, only focused on utilizing hospital service and paid no attention to PHC service. However, previous studies on hospital service utilization helped inform our hypothesis in this study.

 Due to limited studies that explored the impact of the COVID-19 pandemic on PHC service utilization, this study used the outpatient data from 158 PHC institutions in Yinchuan, Ningxia of China, to evaluate the changes in PHC service utilization before, during, and after the COVID-19 pandemic for investigating the effects associated with the COVID-19 pandemic.

## Methods

###  Outpatient Data

 This study included 158 PHC institutions (Figure S1 of Supplementary file 1) from all 380 PHC institutions in Yinchuan, Ningxia, with at least one year outpatient data during 2017-2020. Outpatient data included the number of outpatient visits and outpatient expenditure by date, gender, age, and education level, and the data were aggregated and anonymous (Table S1 and Table S2; see Supplementary file 2). In this study, the outpatient data of the 158 PHC institutions from May 1, 2017 to April 30, 2020 (since this study was started in May 2020) were included in the final analyses.

###  COVID-19 Data

 Data on the number of new COVID-19 cases in Yinchuan, Ningxia and China were collected from the Health Commission of Yinchuan, Health Commission of Ningxia Hui Autonomous Region, and National Health Commission of the People’s Republic of China, which were updated daily during the study period (Table S3 of Supplementary file 2).

###  Classification of Treatment Purposes

 According to the International Classification of Diseases 10th version (ICD-10) code, the outpatient treatment purpose (ie, the primary diagnosis) were classified into 17 types: diseases of the respiratory system (including pneumonia and influenza); diseases of the circulatory system; diseases of the digestive system; endocrine, nutritional and metabolic diseases; diseases of the musculoskeletal system and connective tissue; diseases of the genitourinary system; injury; diseases of the skin and subcutaneous tissue; diseases of the nervous system; diseases of the eye and adnexa; diseases of the ear and mastoid process; certain infectious and parasitic diseases; diseases of the blood and blood-forming organs; neoplasms; oedema, proteinuria and hypertensive disorders in pregnancy, childbirth and the puerperium; mental and behavioral disorders; and pregnancy with abortive outcome. The ICD-10 codes belong to each treatment purpose can be found in Table S4 of Supplementary file 2.

###  Statistical Analysis

 In order to observe the complete influence of the COVID-19 pandemic on the PHC outpatient service, one year before April 30, 2020 (since this study was started in May 2020) was chosen as the observation period (ie, May 1, 2019 to April 30, 2020). Meanwhile, the same periods in two previous years (ie, May 1, 2017 to April 30, 2018 and May 1, 2018 to April 30, 2019) were used as control periods to isolate the association between the COVID-19 pandemic and PHC service utilization. To compare the trend in PHC outpatient service utilization from May 1, 2019 to April 30, 2020 with that in the previous two years, the number of outpatient visits and expenditure were aggregated at the monthly level from May 1, 2017 to April 30, 2020. Since the Chinese Spring Festival was in January or February during the three years we studied (ie, February 16 in 2018; February 5 in 2019; January 25 in 2020), and the COVID-19 appeared in China in December 2019, which almost coincided with this important festival of China, to better preclude the effects of the background levels of different years, we adopted the difference in difference (DID) model (adjusted by gender, age, and education) to analyze the difference in the number of outpatient visits per day, total outpatient expenditure per day, and outpatient expenditure per visit between December 2019 and February 2020 compared with the same periods in the two previous years.

 The total number of outpatient visits and expenditure from January 1, 2020 to April 30, 2020 were also aggregated weekly to evaluate the effects of the COVID-19 outbreak and the government-imposed social restrictions. The weeks were divided as follows: week 1: January 1 to January 7; week 2: January 8 to January 14; week 3: January 15 to January 21; week 4: January 22 to January 28; week 5: January 29 to February 4; week 6: February 5 to February 11; week 7: February 12 to February 18; week 8: February 19 to February 25; week 9: February 26 to March 3; week 10: March 4 to March 10; week 11: March 11 to March 17; week 12: March 18 to March 24; week 13: March 25 to March 31; week 14: April 1 to April 7; week 15: April 8 to April 14; week 16: April 15 to April 21; week 17: April 22 to April 28. January 1 was chosen as the first day of week 1. For one thing, it was the first day of a year; for another, in this way, the first day of week 4 was January 22, when the first COVID-19 case in Ningxia occurred (Table 1) and the following day (ie, January 23) was the first day of Wuhan lockdown. The weekly trends of different treatment purposes were also assessed. Subgroup analyses by gender, age, and education level were performed to examine whether the trends in the number of outpatient visits varied by these demographic characteristics. Wald tests were used to test the heterogeneity in linear coefficients across subgroups. To preclude the effects other than the COVID-19 pandemic on the weekly trend in 2020, the first 17 weeks of data (January 1 was considered as the first day of week 1) in 2018 and 2019 were used as comparisons for the weekly data in 2020. Additionally, the autoregressive integrated moving average (ARIMA) modelling was used to investigate the association between the total number of outpatient visits, expenditure and the number of the last week’s new COVID-19 cases in Yinchuan, Ningxia, and China from week 1 to week 17 in 2020. Subgroup analyses of the ARIMA modelling were done by age, marital status, gender, education, and diagnostic type of the outpatient visits. All statistical analyses were done by Stata 15.0 (StataCorp, College Station, TX, USA).

**Table 1:** Timeline of Implementation of Key COVID-19 Mitigation Measures in Ningxia

**Time**	**Event**
January 22, 2020	The first COVID-19 case in Ningxia was confirmed. The leading group of COVID-19 control and prevention work in Ningxia held the first meeting to arrange the COVID-19 prevention and control work.
January 25, 2020	Initiate the secondary level of public health response.
January 29, 2020	The Health Commission of Yinchuan issued the notice on stopping outpatient service of medical institutions in Yinchuan. From January 31 to February 2, all medical institutions in the city stopped outpatient service (including general outpatient service and expert outpatient service). During the service cessation period, all medical institutions will strengthen the allocation of medical staff in fever clinics and emergency departments. The emergency treatment of cardiovascular, endocrine and some other departments were still available. Consultation and follow-up service were carried out through the internet.
February 3, 2020	Health Commission of Yinchuan began to build the telemedicine platform to provide free online diagnosis and treatment services for Yinchuan citizens.

Abbreviation: COVID-19, coronavirus disease 2019.

## Results

 A total of 1 422 676, 1 736 620, and 2 117 719 individuals visited the PHC institutions included in this study during May 2017 to April 2018, May 2018 to April 2019, and May 2019 to April 2020, respectively (Table 2). Higher proportions of females, those aged 45-64 years, and those with lower education levels were observed in all three periods.

**Table 2:** The Characteristics of Outpatients of Yinchuan PHC Institutions Included in This Study During May 2017 to April 2018, May 2018 to April 2019, and May 2019 to April 2020

**Characteristics**	**May 2017 to April 2018**	**May 2018 to April 2019**	**May 2019 to April 2020**
Total, No. (%)	1 422 676 (100)	1 736 620 (100)	2 117 719 (100)
Gender, No. (%)			
Male	704 977 (49.55)	862 656 (49.67)	1 029 150 (48.60)
Female	717 687 (50.45)	873 952 (50.33)	1 088 519 (51.40)
Age (y), No. (%)			
0-18	264 655 (19.60)	293 774 (18.10)	352 569 (17.35)
19-44	326 461 (24.18)	388 525 (23.94)	442 281 (21.76)
45-64	419 693 (31.09)	527 276 (32.48)	709 649 (34.92)
≥65	339 249 (25.13)	413 659 (25.48)	527 979 (25.98)
Education level, No. (%)			
Elementary school or less	298 856 (42.40)	386 694 (41.62)	485 728 (37.27)
Middle school	224 426 (31.84)	286 636 (30.85)	395 284 (30.33)
High school or vocational school	99 229 (14.08)	134 537 (14.48)	215 168 (16.51)
College/university or above	82 346 (11.68)	121 205 (13.05)	206 926 (15.88)

Abbreviation: PHC, primary healthcare.

###  Outpatient Visits

**Figure 1:**
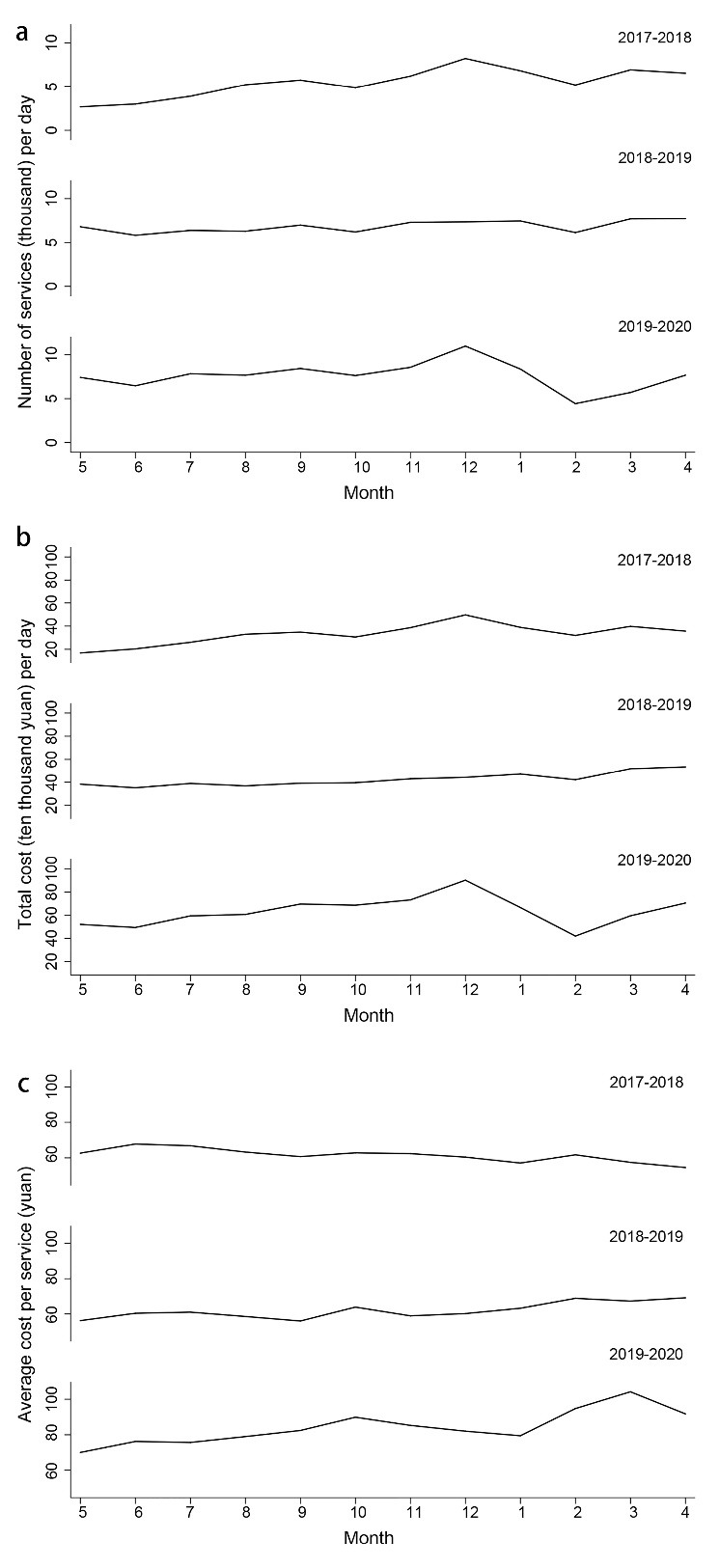
Changes in the Number of Outpatient Visits Per Day (a), Total Outpatient Expenditure Per Day (b) and Outpatient Expenditure Per Visit (c) of Yinchuan PHC Institutions by Month. Abbreviation: PHC, primary healthcare.

 From December 2019 to February 2020, the outpatient visits per day in each month experienced a noticeable decrease (Figure 1a) compared with the two previous years (difference in February versus December in two control periods: -135.35 times, *P* = .056; the difference in February versus December in observation period: -502.56 times, *P*< .001; DID: -367.21 times, *P* = .004).

**Figure 2:**
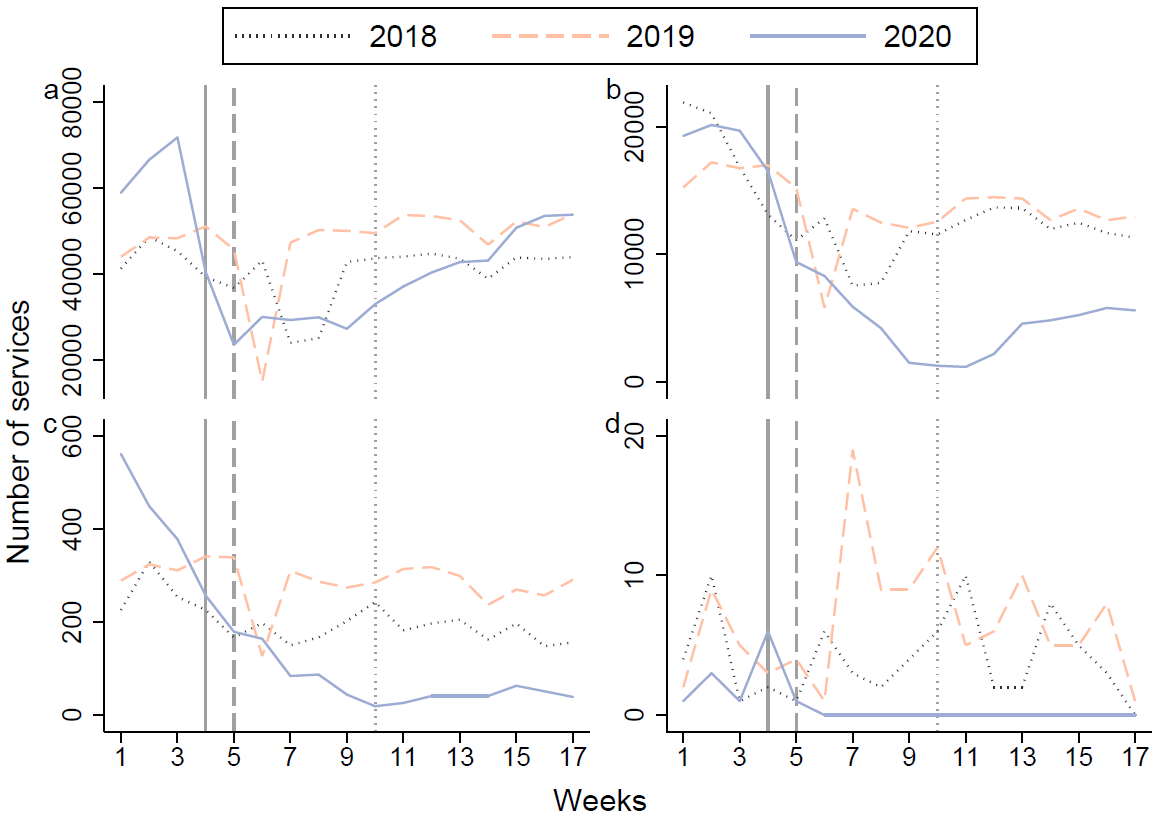
Changes in the Number of Outpatient Visits for All Types of Disease (a), Diseases of the Respiratory System (b), Pneumonia (c), and Influenza (d) of Yinchuan PHC Institutions, by Weeks in 2018, 2019, and 2020. The solid grey vertical line indicated week 4 in 2020 when the first COVID-19 case in Ningxia was confirmed on January 22, and the secondary level of public health response was initiated on January 25. The dashed grey vertical line indicated week 5 in 2020 when Health Commission of Yinchuan issued the notice on stopping outpatient service of medical institutions in Yinchuan on January 29. The dotted grey vertical line indicated week 10 in 2020 when no COVID-19 cases occurred in Yinchuan. Abbreviation: PHC, primary healthcare.

 The weekly trend in 2020 was different in that in the previous two years (Figure 2): the total outpatient visits decreased from week 3 (January 15 to January 21), with the nadir of decreased volume in week 5 (January 29 to February 4), followed by a steady rise after that. For most treatment purposes, the outpatient visits experienced a similar trend except for diseases of the respiratory system and pregnancy with abortive outcomes (Figure S2 of Supplementary file 1). As for the number of outpatient visits for diseases of the respiratory system, it dropped from week 2 (January 8 to January 14) to week 11 (March 11 to March 17), with the rebound delayed by six weeks when there have been no new cases in Ningxia for two weeks (Table S3 of Supplementary file 2). However, for pneumonia, the outpatient visits kept declining until week 17 (April 22 to April 28), and almost no outpatient visits for influenza during the same period. Besides, the number of outpatients visits for pregnancy with abortive outcomes kept steady although small.

**Figure 3:**
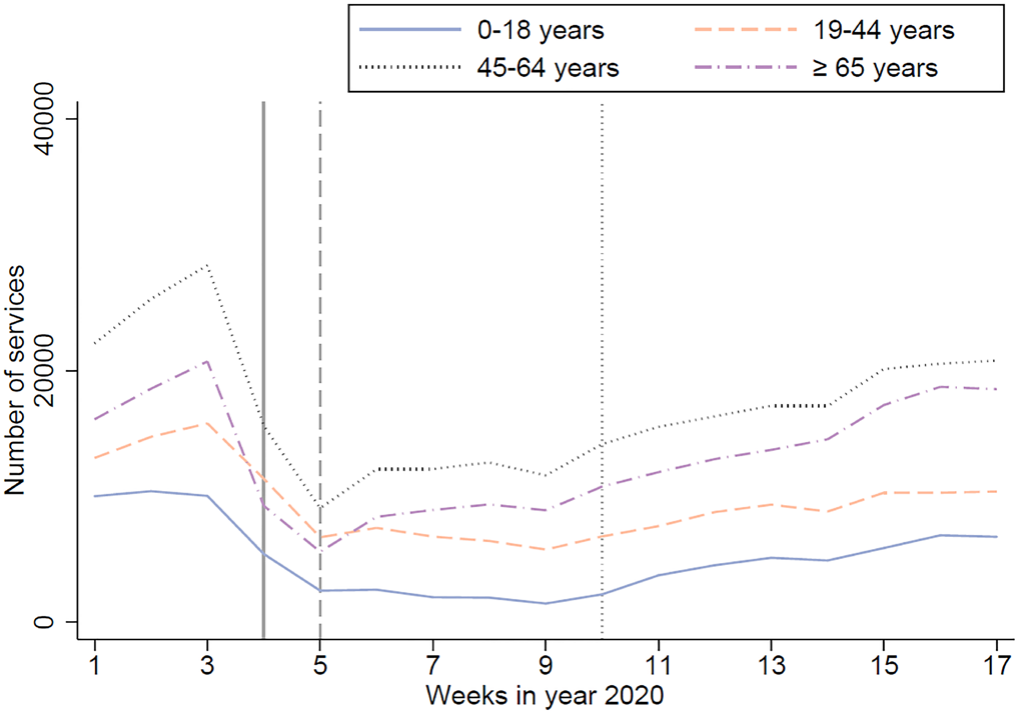
Age Differences in Changes in the Number of Outpatient Visits of Yinchuan PHC Institutions in Weeks of 2020. The solid grey vertical line indicated week 4 when the first COVID-19 case in Ningxia was confirmed on January 22, and the secondary level of public health response was initiated on January 25. The dashed grey vertical line indicated week 5 when Health Commission of Yinchuan issued the notice on stopping outpatient service of medical institutions in Yinchuan on January 29. The dotted grey vertical line indicated week 10 when no COVID-19 cases occurred in Yinchuan. Abbreviation: PHC, primary healthcare.

 The declining trend in different genders from week 3 to week 5 was similar (Wald test: *P* = .265), but the females displayed a steeper rebound (*P* = .010) from week 5 to week 17 (Figure S3 of Supplementary file 1**)**. Those with lower education levels displayed a steeper decrease from week 3 to week 5 (*P*< .001) and rebound from week 5 to week 17 (*P*< .001) compared with those with higher education levels (Figure S4 of Supplementary file 1). The middle-aged and elderly population (ie, ≥45 years) displayed a steeper decrease from week 3 to week 5 (*P*< .001) and rebound from week 5 to week 17 (*P*< .001) compared with those aged ≤44 years (Figure 3). According to the results of ARIMA modelling, the total number of outpatient visits per week was negatively associated with the number of the last week’s new COVID-19 cases in China (Coefficient = -0.99, *P*< .001), but not with that in Yinchuan (Coefficient = -355.58, *P* = .791) and Ningxia (Coefficient = 52.47, *P* = .919). The results of the subgroup analyses of the ARIMA modelling by age, marital status, gender, education, and diagnostic type of the outpatient visits can be seen in Table S5 of Supplementary file 2.

###  Outpatient Expenditure

 The outpatient expenditure per day in each month dropped larger from December 2019 to February 2020 than in two previous years (Figure 1b, difference in February versus December in two control periods: -5.47 thousand yuan, *P* = .377; difference in February versus December in observation period: -25.39 thousand yuan, *P* = .006; DID: -19.92 thousand yuan, *P* = .068). According to Figure 1c, the outpatient expenditure per visit raised more from December 2019 to February 2020 than in two previous years (difference in February versus December in two control periods: 2.42 yuan per visit, *P* = .488; difference in February versus December in observation period: 21.48 yuan per visit, *P*< .001; DID: 19.06 thousand yuan, *P* = .003).

 According to the results of ARIMA modelling, the total outpatient expenditure (million yuan) per week was negatively related to the number of the last week’s new COVID-19 cases in Yinchuan (Coefficient = -0.18, *P*< .001), Ningxia (Coefficient = -0.09, *P*< .001), and China (Coefficient = -0.00008, *P*< .001).

## Discussion

 Using the PHC outpatient service data, we evaluated the impact of the COVID-19 pandemic on PHC, which is the basis of the healthcare system in China.^16^ There were three main findings in this study. First, the decline and rebound of the total outpatient volume during the onset of the COVID-19 pandemic in 2020 were larger than in the same period of the previous two years, and the pattern in the rebound for the diseases of the respiratory system was different. Second, the decline and rebound of the outpatient service utilization in the middle-aged and elderly were steeper than in those younger. Third, there was a negative correlation between outpatient volume and the number of the last week’s new COVID-19 cases.

 The total outpatient volume declined more from December 2019 to February 2020 than in the same period of two previous years, and also rebounded more evident after February 2020 than in the same period of two previous years. The possible explanations for the steeper decline and rebound in 2020 are as follows. First, several patients may defer their healthcare to reduce social contact and avoid healthcare institutions for fear of COVID-19 contagion.^6-8,10,12^ Second, the government-imposed restrictions may have a significant impact on the reduction. From January 31 to February 2, which lie in week 5 of 2020 (ie, the nadir of outpatient volume), outpatient service in Yinchuan was stopped. Also, most medical institutions restarted outpatient service in early March, which coincided with the rebound after week 9. The decline and rebound during and after government-imposed restrictions were observed in the United States as well.^10^ Third, internet hospitals are widely adopted in Yinchuan during the COVID-19 pandemic, which may account for part of reductions in in-person PHC visits.^6^ For China, internet hospitals is a novel way to deliver outpatient service, by which patients can consult a doctor in a top-level hospital from a big city through the internet.^17^ Comparing with the studies from other countries, the response rate of healthcare utilization to the pandemic was similar; the healthcare utilization declined at the beginning of the pandemic and rebounded soon after the initial peak of the pandemic.^7,18^ Also, in this study, we found a negative association between the total number of outpatient visits and the number of the last week’s new COVID-19 cases. It indicates that the public’s prediction of the infection risk is affected by the severity of the pandemic. Thus, besides the aforementioned unquantifiable factors, the severity of the COVID-19 pandemic was also a significant quantifiable factor.^19^ But it was reported that in-person healthcare utilization would not increase the risk of infection if it was safely done.^20^ Therefore, during the pandemic, the public should be given enough confidence and protection guidance to obtain the necessary healthcare in time, then helping avoid adverse clinical outcomes.^10^

 Classified by treatment purposes, outpatient service for pregnancy with abortive outcome, a type of nonelective healthcare, was less influenced by the pandemic, similar to the findings in previous studies from other countries.^6,9,10^ As for the disease of the respiratory systems, the reduction was largest compared to other types of diseases, which can be found in previous studies.^7,21^ Also, the rebound for it was six weeks later than most other types of diseases. The apparent reduction and delayed rebound of outpatient visits for the disease of the respiratory systems may be because the prevention and control measures recommended during the COVID-19 pandemic such as social distancing, face masks, and limited social gatherings can also help curb the spread of other infectious respiratory diseases. In a study from the United States, there were steep declines in prescriptions of amoxicillin and azithromycin (used for upper respiratory tract infection symptoms) during the COVID-19 pandemic when excluding the impact of expected seasonal declines.^22^ In Singapore, fewer non-COVID-19-related respiratory disease hospital admissions due to social distancing and the use of masks were also observed.^23^ However, we also found that the outpatient expenditure per visit was on a larger increase during the same period. According to a study in the United States, there were dramatic reductions in the use of preventive and elective care during the first two months of the COVID-19 pandemic, but not in the use of nonelective care and prescription drugs.^6^ Therefore, the increasing outpatient expenditure per visit may be because patients were prescribed more drugs at a single visit to reduce the frequency of visiting PHC institutions.

 The decline and rebound of the outpatient service utilization in the middle-aged and elderly were steeper than in those younger. As for the vulnerability of the middle-aged and elderly population, it was also observed in the United States as the decrease in non-COVID-19 hospital admissions was greater among the population aged over 70 than those younger than 50.^10^ The steeper reduction in the initial months of the pandemic maybe because of the fact that advanced age is associated with the increased risk^24^ and seriousness^25^ of COVID-19 which was widely published by the media. Therefore, given the added concern and pressure about getting infected, middle-aged and elderly people may be reluctant to leave their homes to visit doctors during the lockdown period.^9^ The steeper rebound followed the decline in the middle-aged and elderly population reflected a large number of delayed outpatient visits. For one thing, it may be because elderly people may have difficulties in the utilization of internet hospitals.^26^ For another, the “long prescription” policy implemented during the pandemic in China may also play a role. The “long prescription” policy was to encourage a reasonable increase in the dosage of a single prescription to reduce the frequency of visits for dispensing; and for patients with chronic diseases such as hypertension and diabetes, the dosage of a prescription can be extended to three months.^27^ Since one of the core purposes of PHC service in China is chronic disease management which is mainly for the middle-aged and elderly population,^28,29^ the demand for medical service of the middle-aged and elderly population can be largely reduced during the initial months of the COVID-19 pandemic. Therefore, in the formulation of the emergency strategy, vulnerable populations such as the older population should be given extra and distinct attention. Although some measures have been proposed in the guideline for healthcare institutions to provide medical and health services for the older patients with chronic diseases during the prevention and control period of COVID-19 pandemic in China,^30^ including long-term prescription, doctor visiting at home, and prescribing medicine by family members. According to the suggestions of World Health Organization (WHO) and the experience in other countries, there are still some measures can be taken. First, improving digital literacy of older population is important. Older population should be taught by their family members or the community doctors to use telemedicine such as mobile apps to receive information and seek help from PHC providers even when staying at home, which has been also emphasized in a systematic review exploring the potentials of telehealth during the COVID-19 pandemic.^31^ Second, the government should ensure that the family members and caregivers of the older population are supported as a priority and given enough sources to help satisfy the medical needs of the older people. Third, social support such as voluntary work plays an important role as well, which can help ensure the nutritious food and medicine for the older people. Furthermore, older people should be told what to do to keep healthy or to cope with illness during the pandemic through public media or community doctors.^32^ Also, based on hospital systems in China, constructing a regional medical consortium (ie, it is composed of tertiary hospitals, secondary hospitals and PHC institutions within a certain region, in which all cooperative units support dual referral systems and appointments) can be a good way to enhance the efficiency in the utilization of medical resources, especially for the older people and those with chronic conditions.^33,34^

 This study firstly evaluated the impact of the COVID-19 pandemic on the utilization of PHC, which functions as the basis of the Chinese healthcare system, based on the outpatient data from 158 PHC institutions in Yinchuan, Ningxia of China. The same periods in two previous years were used as control periods to isolate the association between the COVID-19 pandemic and PHC service utilization. However, there were still some limitations in this study. First, Yinchuan was not a high-risk area during the COVID-19 pandemic; therefore, this study’s findings may not reflect the situation in high-risk areas. Nevertheless, in relatively low-risk Yinchuan, the significant impact from the COVID-19 pandemic was already found, let alone the impact on high-risk areas, where more serious delayed seeking for healthcare service could be reasonably speculated. Second, we cannot know the trend in the utilization of online healthcare services during the same period to evaluate the decline in offline service that the online service can offset; therefore, further studies are warranted. Third, due to the limit of data availability, we cannot analyze the trends in utilization of prevention services, such as vaccination and physical examination, which are of great importance for clinical practice and policy-making. Fourth, only outpatient service was considered in this study. However, since PHC is aimed to provide affordable, accessible and available services to achieve universal health coverage, outpatient service takes the majority of its usual business, with a small proportion of inpatient care.^14,16^

## Conclusion

 The large decline in PHC outpatient service utilization during the initial two months of the COVID-19 pandemic in Yinchuan indicated the significant impact of the COVID-19 pandemic on medical services. Since PHC is the foundation of the healthcare system in China and in most other developing countries, measures (eg, meeting necessary healthcare under reasonable protection, focusing on the vulnerable populations, “long prescription” policy) should be taken to make it play an important role in coping with crisis and helping relieve the burden of hospital care.

## Acknowledgements

 The authors thank prof. Yinzi Jin (Department of Global Health, School of Public Health, Peking University, Beijing, China) and Xu Qin (School of Education, University of Pittsburgh, Pittsburgh, PA, USA) for providing help in statistical methods.

## Ethical issues

 This study was approved by the ethical review committee of Peking University First Hospital (IRB. No: 202001001), and the requirement for informed consent was waived.

## Competing interests

 Authors declare that they have no competing interests.

## Authors’ contributions

 LX, SW, and HX took the responsibility of study conception and design. LZ and GL contributed to data collection and assembly. LX and GL contributed to data verification. LX, LZ, JZ, WY, GL, SZ, SW, and HX contributed to data interpretation. LX contributed to statistical analysis. LX was responsible for manuscript draft. All authors contributed to manuscript review and revision, and approval of submission.

## Funding

 This work was supported by the Ningxia Provincial Natural Science Foundation [grant number 2020AAC03501] and Innovation Fund for Outstanding Doctoral Candidates of Peking University Health Science Center (China). The funder had no role in the design of the study and collection, analysis, and interpretation of data and in writing the manuscript.

## Supplementary files

Supplementary file 1 contains Figures S1-S4.

Supplementary file 2 contains Tables S1- S5.
