# Supplementary material for: Impact of the COVID-19 Pandemic on Outpatient Service in Primary Healthcare Institutions: An Inspiration From Yinchuan of China
Source: Int J Health Policy Manag. 2021 Aug 31;11(9):1926–33. doi: 10.34172/ijhpm.2021.119 (PMC9808251; doi:10.34172/ijhpm.2021.119)
Supplement: Supplementary file 1 — contains Figures S1-S4. [file ijhpm-11-1926-s001.pdf]

Article title: Impact of the COVID-19 Pandemic on Outpatient Service in Primary Healthcare Institutions: An Inspiration From Yinchuan of China

Journal name: International Journal of Health Policy and Management (IJHPM)

Authors' information: Lu Xu[1], Lin Zhuo[2], Jie Zhang[3], Wu Yang[3], Guozhen Liu[4], Siyan Zhan[1,2,5], Shengfeng Wang[1]*, Huijie Xiao[6*]

[1]Department of Epidemiology and Biostatistics, School of Public Health, Peking University, Beijing, China.

[2]Research Center of Clinical Epidemiology, Peking University Third Hospital, Beijing, China.

[3]Maternal and Child Health Care Hospital of Ningxia Hui Autonomous Region, Yinchuan, China.

[4]Peking University Health Information Technology Co. Ltd, Beijing, China.

[5]Center for Intelligent Public Health, Institute for Artificial Intelligence, Peking University, Beijing, China.

[6]Department of Paediatrics, Peking University First Hospital, Beijing, China.

(*corresponding author: Shengfeng Wang; Email: shengfeng1984@126.com & Huijie Xiao; Email: 13810696936@163.com)

Supplementary file 1. Contains Figures S1-S4

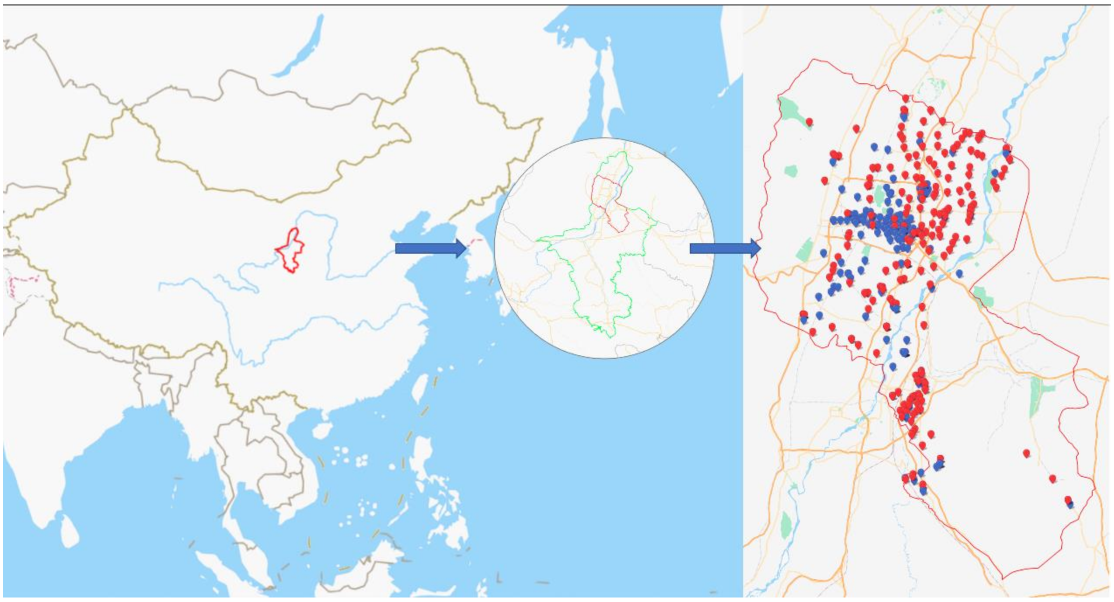

Figure S1. The distribution of 380 Yinchuan primary health care institutions

Note: the blue dots are 158 Yinchuan primary health care institutions included in this study, while the red dots were those not included in this study due to unavailable data.

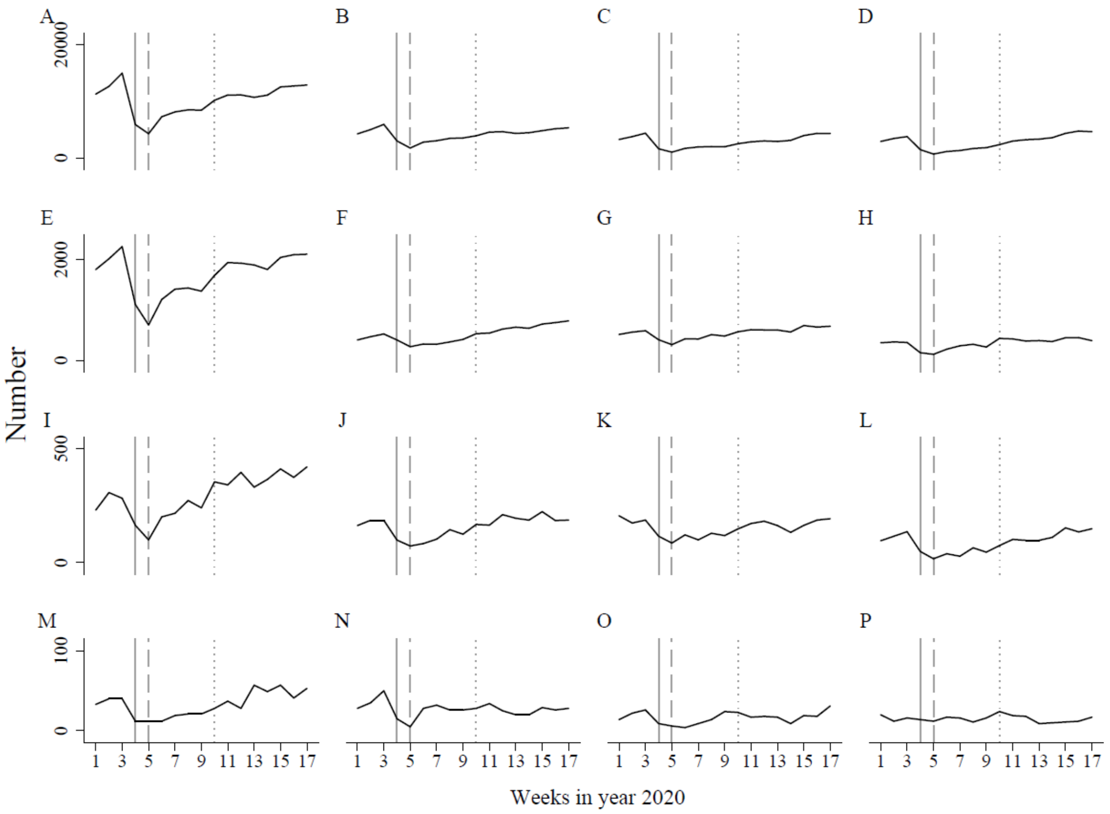

Figure S2. Changes in the number of outpatient visits of Yinchuan primary health care institutions in weeks of 2020, by disease type

A: Diseases of the circulatory system; B: Diseases of the digestive system; C: Endocrine, nutritional and metabolic diseases; D: Diseases of the musculoskeletal system and connective tissue; E: Diseases of the genitourinary system; F: Injury; G: Diseases of the skin and subcutaneous tissue; H: Diseases of the nervous system; I: Diseases of the eye and adnexa; J: Diseases of the ear and mastoid process; K: Certain infectious and parasitic diseases; L: Diseases of the blood and blood-forming organs; M: Neoplasms; N: Oedema, proteinuria and hypertensive disorders in pregnancy, childbirth and the puerperium; O: Mental and behavioral disorders; P: Pregnancy with abortive outcome

Note: Week 1: January 1 to January 7; Week 2: January 8 to January 14; Week 3: January 15 to January 21; Week 4: January 22 to January 28; Week 5: January 29 to February 4; Week 6: February 5 to February 11; Week

7: February 12 to February 18; Week 8: February 19 to February 25; Week 9: February 26 to March 3; Week 10: March 4 to March 10; Week 11: March 11 to March 17; Week 12: March 18 to March 24; Week 13: March 25 to March 31; Week 14: April 1 to April 7; Week 15: April 8 to April 14; Week 16: April 15 to April 21; Week 17: April 22 to April 28. The solid grey vertical line indicated week 4 when the first COVID-19 case in Ningxia was confirmed on January 22, and the secondary level of public health response was initiated on January 25. The dashed grey vertical line indicated week 5 when Health Commission of Yinchuan issued the notice on stopping outpatient service of medical institutions in Yinchuan on January 29. The dotted grey vertical line indicated week 10 when no COVID-19 cases occurred in Yinchuan.

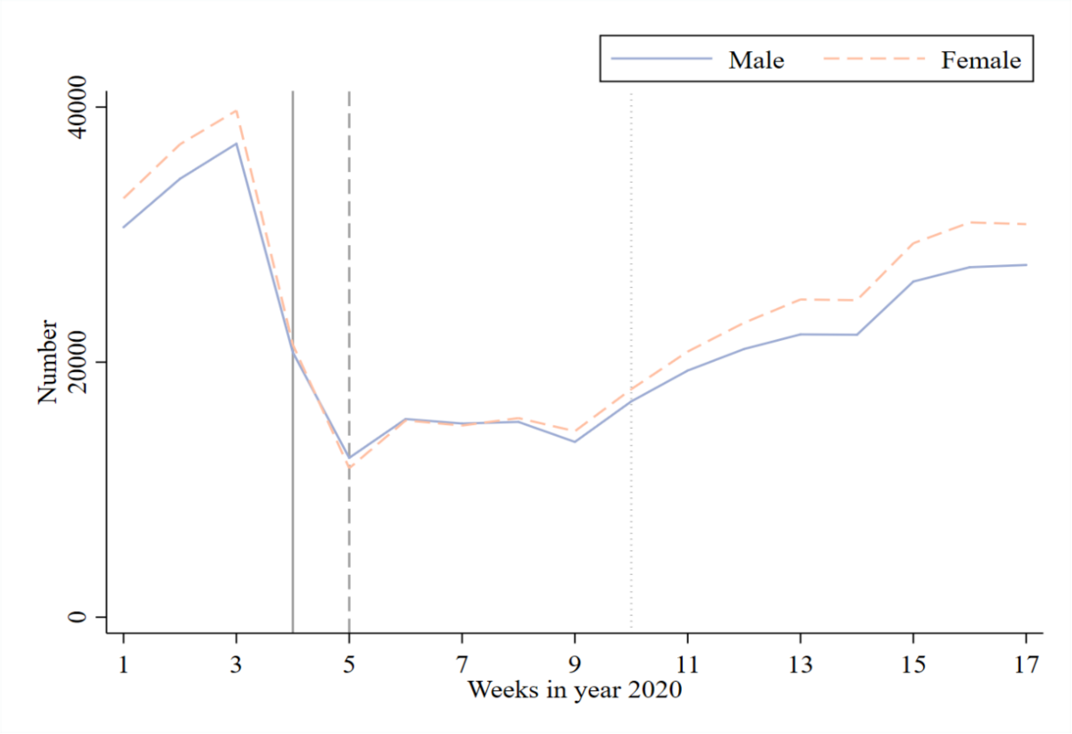

Figure S3. Sex differences in changes in the number of outpatient visits of Yinchuan primary health care institutions in weeks of 2020

Note: Week 1: January 1 to January 7; Week 2: January 8 to January 14; Week 3: January 15 to January 21; Week 4: January 22 to January 28; Week 5: January 29 to February 4; Week 6: February 5 to February 11; Week 7: February 12 to February 18; Week 8: February 19 to February 25; Week 9: February 26 to March 3; Week 10: March 4 to March 10; Week 11: March 11 to March 17; Week 12: March 18 to March 24; Week 13: March 25 to March 31; Week 14: April 1 to April 7; Week 15: April 8 to April 14; Week 16: April 15 to April 21; Week 17: April 22 to April 28. The solid grey vertical line indicated week 4 when the first COVID-19 case in Ningxia was confirmed on January 22, and the secondary level of public health response was initiated on January 25. The dashed grey vertical line indicated week 5 when Health Commission of Yinchuan issued the notice on stopping outpatient service of medical institutions in Yinchuan on January 29. The dotted grey vertical line indicated week 10 when no COVID-19 cases occurred in Yinchuan.

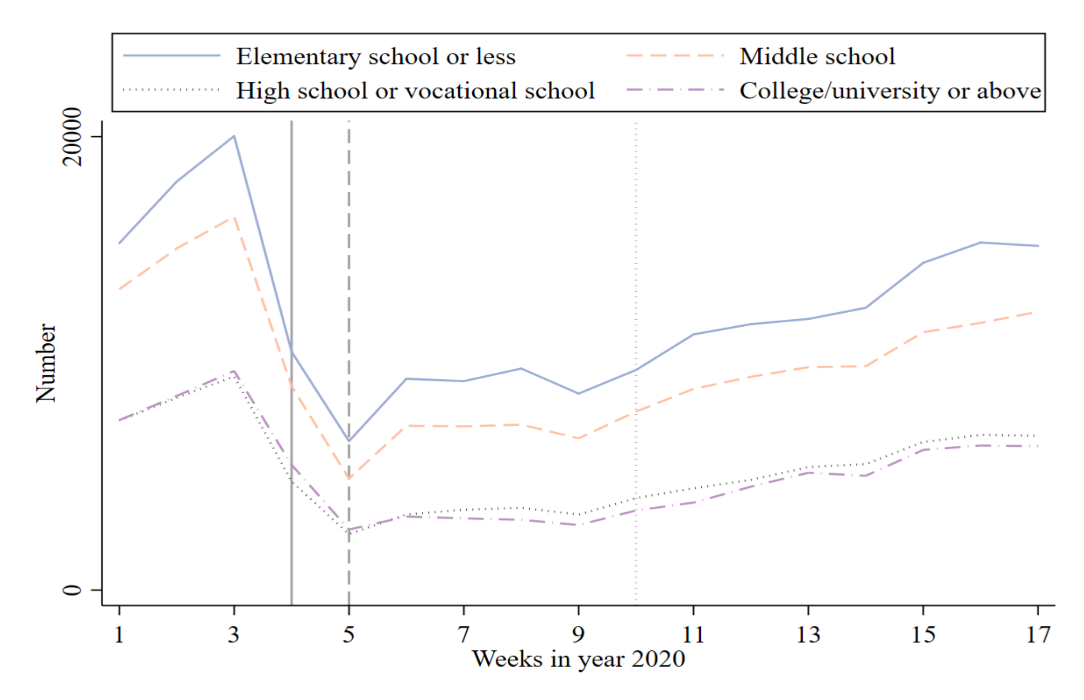

Figure S4. Changes in the number of outpatient visits of Yinchuan primary health care institutions in weeks of 2020, by education level

Note: Week 1: January 1 to January 7; Week 2: January 8 to January 14; Week 3: January 15 to January 21; Week 4: January 22 to January 28; Week 5: January 29 to February 4; Week 6: February 5 to February 11; Week 7: February 12 to February 18; Week 8: February 19 to February 25; Week 9: February 26 to March 3; Week 10: March 4 to March 10; Week 11: March 11 to March 17; Week 12: March 18 to March 24; Week 13: March 25 to March 31; Week 14: April 1 to April 7; Week 15: April 8 to April 14; Week 16: April 15 to April 21; Week 17: April 22 to April 28. The solid grey vertical line indicated week 4 when the first COVID-19 case in Ningxia was confirmed on January 22, and the secondary level of public health response was initiated on January 25. The dashed grey vertical line indicated week 5 when Health Commission of Yinchuan issued the notice on stopping outpatient service of medical institutions in Yinchuan on January 29. The dotted grey vertical line indicated week 10 when no COVID-19 cases occurred in Yinchuan.
