# Supplementary material for: Impact of the COVID-19 Pandemic on Outpatient Service in Primary Healthcare Institutions: An Inspiration From Yinchuan of China
Source: Int J Health Policy Manag. 2021 Aug 31;11(9):1926–33. doi: 10.34172/ijhpm.2021.119 (PMC9808251; doi:10.34172/ijhpm.2021.119)
Supplement: Supplementary file 2 — contains Tables S1- S5. [file ijhpm-11-1926-s002.pdf]

Article title: Impact of the COVID-19 Pandemic on Outpatient Service in Primary Healthcare Institutions: An Inspiration From Yinchuan of China

Journal name: International Journal of Health Policy and Management (IJHPM)

Authors' information: Lu Xu[1], Lin Zhuo[2], Jie Zhang[3], Wu Yang[3], Guozhen Liu[4], Siyan Zhan[1,2,5], Shengfeng Wang[1]*, Huijie Xiao[6*]

[1]Department of Epidemiology and Biostatistics, School of Public Health, Peking University, Beijing, China.

[2]Research Center of Clinical Epidemiology, Peking University Third Hospital, Beijing, China.

[3]Maternal and Child Health Care Hospital of Ningxia Hui Autonomous Region, Yinchuan, China.

[4]Peking University Health Information Technology Co. Ltd, Beijing, China.

[5]Center for Intelligent Public Health, Institute for Artificial Intelligence, Peking University, Beijing, China.

[6]Department of Paediatrics, Peking University First Hospital, Beijing, China.

(*corresponding author: Shengfeng Wang; Email: shengfeng1984@126.com & Huijie Xiao; Email: 13810696936@163.com)

Supplementary file 1. Contains Tables S1- S5

Table S1. The sample data of outpatient visits

Date	Sex	Age (years)	Marriage	Education	ICD 10	Number of outpatient visits
Distinct values:	Distinct	0;	Distinct	Distinct values:	Distinct values:	
2017-01-01;	values:	1;	values:	Elementary school or less;	A00-B99;	
2017-01-02;	Male;	2;	Married;	Middle school;	C00-D48;	
2017-01-03;	Female;	3;	Unmarried	High school or vocational school;	D50-D89;	
…	Other	4;	;	College/university or above	E00-E90;	
2017-02-01;		5;	Other		F00-F99;	
2017-02-02;		6;			G00-G99;	
2017-02-03;		7;			H00-H59;	
…		8;			H60-H95;	
…		9;			I00-I99;	
2018-01-01;		10;			J00-J99;	
2018-01-02;		11;			J09-J11;	
2018-01-03;		12;			J12-J18;	
…		13;			J19-J99;	
…		14;			K00-K93;	

2019-01-01;	15-18;	L00-L99;
2019-01-02;	19;	M00-M99;
2019-01-03;	20-24;	N00-N99;
…	25-29;	O00-O08;
…	30-34; 35-	O10-O16;
2020-01-01;	39;	O20-O29;
2020-01-02;	40-44;	O30-O48;
2020-01-03;	45-49;	O60-O75;
…	50-54;	O80-O84;
…	55-59;	O85-O92;
2020-04-28;	60-64;	O94-O99;
2020-04-29;	65-69;	P00-P96;
2020-04-30	70-74;	Q00-Q99;
	75-79;	S00-T35+V01-V99+W00-X19+X30-X39+Y20-
	≥80	Y31;
		T36-T65+X20-X29+X40-X49+Y10-Y19;
		U04;
		V01-V99+Y32;
		X60-X84;

					Other;	
					Any type	

Table S2. The sample data of outpatient expenditure

**Date**	**Sex**	**Age (years)**	**Marriage**	**Education**	**Number of outpatient visits**	**Total of outpatient expenditure**
Distinct values:	Distinct	0;	Distinct	Distinct values:		
2017-01-01;	values:	1;	values:	Elementary school or less;		
2017-01-02;	Male;	2;	Married;	Middle school;		
2017-01-03;	Female;	3;	Unmarried;	High school or vocational school;		
…	Other	4;	Other	College/university or above		
2017-02-01;		5;				
2017-02-02;		6;				
2017-02-03;		7;				
…		8;				
…		9;				
2018-01-01;		10;				
2018-01-02;		11;				

2018-01-03;	12;
…	13;
…	14;
2019-01-01;	15-18;
2019-01-02;	19;
2019-01-03;	20-24;
…	25-29;
…	30-34; 35-39;
2020-01-01;	40-44;
2020-01-02;	45-49;
2020-01-03;	50-54;
…	55-59;
…	60-64;
2020-04-28;	65-69;
2020-04-29;	70-74;
2020-04-30	75-79;
	≥80

Table S3. Number of new COVID-19 cases in Yinchuan, Ningxia, and China in weeks of 2020

Week	Yinchuan	Ningxia	China
1	0	0	0
2	0	0	41
3	0	0	399
4	7	11	5534
5	13	22	18389
6	11	24	20367
7	2	13	29549
8	0	0	3911
9	3	4	2232
10	0	0	533
11	0	0	196
12	0	0	695
13	0	0	785
14	0	0	526
15	0	0	588
16	0	0	542
17	0	0	82

Table S4. The classifications of treatment purposes according to the International Classification of Diseases 10th version (ICD-10)

Treatment purposes	ICD-10 codes
Diseases of the respiratory system	J00-J99
Pneumonia	J12-J18
Influenza	J09-J11
Diseases of the circulatory system	I00-I99
Diseases of the digestive system	K00-K93
Endocrine, nutritional and metabolic diseases	E00-E90
Diseases of the musculoskeletal system and connective tissue	M00-M99
Diseases of the genitourinary system	N00-N99
Injury	S00-T35+ V01-V99+W00-X19+X30-X39+Y20-Y31
Diseases of the skin and subcutaneous tissue	L00-L99
Diseases of the nervous system	G00-G99
Diseases of the eye and adnexa	H00-H59
Diseases of the ear and mastoid process	H60-H95
Certain infectious and parasitic diseases	A00-B99
Diseases of the blood and blood-forming organs	D50-D89
Neoplasms	C00-D48
Oedema, proteinuria and hypertensive disorders in pregnancy, childbirth and the puerperium	O10-O16

Mental and behavioral disorders	F00-F99
Pregnancy with abortive outcome	O00-O08

Table S5. Results of the subgroup analyses of the autoregressive integrated moving average modelling for the association between PHC outpatient service use per week and the last week's new COVID-19 cases in China, by age, sex, education, marital status, and diagnostic type of the outpatient visits

Subgroup	The total number of outpatient visits per week		The total outpatient expenditure (million yuan) per week	
Age	Coefficient	P-value	Coefficient	P-value
0-18	0.006	0.503	-0.00002	<0.001
19-44	-0.168	0.414	-0.00002	<0.001
45-64	-0.311	<0.001	-0.00001	0.018
≥65	-0.273	<0.001	-0.00001	0.079
Sex				
Male	-0.448	<0.001	-0.00003	<0.001
Female	-0.545	<0.001	-0.00004	<0.001
Education				
Elementary school or less	-0.192	0.001	-0.00001	0.023
Middle school	-0.177	<0.001	-0.00001	0.023
High school or vocational school	-0.119	<0.001	-0.000003	0.313
College/university or above	0.005	0.693	-0.00001	0.001
Marital status				
Married	-0.515	<0.001	-0.00002	0.038
Unmarried	0.029	<0.001	-0.000005	0.005

Diagnostic type of the outpatient visits				
Diseases of the respiratory system	0.079	0.028	NA	NA
Diseases of the circulatory system	-0.145	0.003	NA	NA
Diseases of the digestive system	-0.062	0.003	NA	NA
Endocrine, nutritional and metabolic diseases	-0.061	<0.001	NA	NA
Diseases of the musculoskeletal system and connective tissue	-0.005	0.282	NA	NA
Diseases of the genitourinary system	-0.022	0.002	NA	NA
Injury	0.004	<0.001	NA	NA
Diseases of the skin and subcutaneous tissue	0.001	0.146	NA	NA
Diseases of the nervous system	0.002	0.238	NA	NA
Diseases of the eye and adnexa	0.002	0.040	NA	NA
Diseases of the ear and mastoid process	-0.003	0.029	NA	NA
Certain infectious and parasitic diseases	0.0004	0.862	NA	NA
Diseases of the blood and blood-forming organs	0.001	0.305	NA	NA

* No available data in weekly outpatient expenditure of different diagnostic types.
